# Go-6976 Reverses Hyperglycemia-Induced Insulin Resistance Independently of cPKC Inhibition in Adipocytes

**DOI:** 10.1371/journal.pone.0108963

**Published:** 2014-10-15

**Authors:** Katherine A. Robinson, Krisztina Hegyi, Yusuf A. Hannun, Maria G. Buse, Jaswinder K. Sethi

**Affiliations:** 1 The Department of Clinical Biochemistry, University of Cambridge Metabolic Research Laboratories, Institute of Metabolic Science, Addenbrooke's Hospital, Cambridge, United Kingdom; 2 The Departments of Biochemistry and Molecular Biology and Medicine, Division Diabetes, Endocrinology and Medical Genetics, Medical University of South Carolina, Charleston, South Carolina, United States of America; 3 The Department of Medicine and the Stony Brook Cancer Center, Stony Brook University, Stony Brook, New York, United States of America; GDC, Germany

## Abstract

Chronic hyperglycemia induces insulin resistance by mechanisms that are incompletely understood. One model of hyperglycemia-induced insulin resistance involves chronic preincubation of adipocytes in the presence of high glucose and low insulin concentrations. We have previously shown that the mTOR complex 1 (mTORC1) plays a partial role in the development of insulin resistance in this model. Here, we demonstrate that treatment with Go-6976, a widely used “specific” inhibitor of cPKCs, alleviates hyperglycemia-induced insulin resistance. However, the effects of mTOR inhibitor, rapamycin and Go-6976 were not additive and only rapamycin restored impaired insulin-stimulated AKT activation. Although, PKCα, (but not –β) was abundantly expressed in these adipocytes, our studies indicate cPKCs do not play a major role in causing insulin-resistance in this model. There was no evidence of changes in the expression or phosphorylation of PKCα, and PKCα knock-down did not prevent the reduction of insulin-stimulated glucose transport. This was also consistent with lack of IRS-1 phosphorylation on Ser-24 in hyperglycemia-induced insulin-resistant adipocytes. Treatment with Go-6976 did inhibit a component of the mTORC1 pathway, as evidenced by decreased phosphorylation of S6 ribosomal protein. Raptor knock-down enhanced the effect of insulin on glucose transport in insulin resistant adipocytes. Go-6976 had the same effect in control cells, but was ineffective in cells with Raptor knock-down. Taken together these findings suggest that Go-6976 exerts its effect in alleviating hyperglycemia-induced insulin-resistance independently of cPKC inhibition and may target components of the mTORC1 signaling pathway.

## Introduction

Insulin resistance is a common pathological condition defined as the reduced ability of insulin to produce its biological effects. Clinically, it refers to the reduced effectiveness of insulin to lower plasma glucose. Insulin resistance is a major feature of Type 2 diabetes and is commonly associated with numerous other conditions, *e.g.* obesity, the metabolic syndrome, polycystic ovary syndrome, glucocorticoid excess, *etc.* The molecular basis of insulin resistance is currently incompletely understood, but in each of these conditions is likely to be multi-factorial, being induced by glucotoxicity, lipotoxicity, and hormones. In contrast, the insulin resistance that accompanies uncontrolled Type 1 diabetes is primarily attributed to glucotoxicity and is reversible with insulin therapy *in vivo* [1], [2]. However, the mechanistic basis of hypergylcemia-induced insulin resistance is controversial. Its expression appears to be tissue/cell-type specific and may differ from that caused by lipotoxicity.

We have studied the mechanism(s) of insulin resistance in an *in vitro* model of glucotoxicity in 3T3-L1 adipocytes in some detail [3], [4]. This represents a model of ‘fasting hyperglycemia-induced insulin resistance’ as is observed in diabetes. Under control conditions (*i.e.* after incubation in 5 mM glucose), adipocytes are exquisitely insulin responsive - as assessed by the insulin stimulated glucose uptake. Chronic preincubation in either high glucose (25 mM) or in the presence of a relatively low dose of insulin (0.6 nM) alone does not alter the insulin-stimulated response. However, chronic preincubation with both high glucose and low insulin causes a 40–60% impairment of subsequent insulin-stimulated glucose transport. This state of insulin resistance is not accompanied by changes in the expression of the glucose transporters, GLUT1 and GLUT4 and the proximal insulin signaling cascade appears to be intact, as judged by the insulin response of insulin receptor substrate-1 (IRS-1)-bound phosphatidylinositol (PI) 3-kinase activity [3]. However, in this model there is a block downstream of PI3-kinase activation, which is manifested by markedly impaired activation of protein kinase B/Akt [5].

Since we had evidence that flux through the hexosamine synthesis pathway was increased by exposure to high glucose, we explored the possibility that the insulin resistance reflected enhanced O-GlcNAc modification of some regulatory proteins. However, increased expression of the enzyme which removes O-GlcNAc from proteins or >90% knock-down of O-GlcNAc transferase did not affect the development of insulin resistance in our model, suggesting that other pathways certainly played a role [6]. Similarly, we found no evidence to suggest that enhanced superoxide production caused the insulin resistance in this model [7].

We discovered that basal PtdIns(3,4,5)P_3_ was unaltered in control and insulin resistant cells, however, following insulin stimulation, the response was diminished by 34% in the latter. Concomitantly, the amount of PTEN protein, a lipid phosphatase, which dephosphorylates PtdIns(3,4,5)P_3_ in the 3-position was significantly and specifically increased in the insulin resistant cells. Treatment with rapamycin, a specific inhibitor of mTORC1, inhibited increased PTEN expression and partially restored insulin-stimulated glucose transport and Akt activation to the insulin resistant cells. Thus, we have evidence that at least one mechanism, which causes insulin resistance in our model, involves the mTOR pathway [7].

Given that blocking mTORC1 restored the insulin resistance only partially, we continued the search for other pathways, which may be involved. Protein kinase C (PKC) seemed to be a logical candidate, particularly because it is known to be activated under some conditions by exposure to high glucose [8]. This hypothesis was supported by initial experiments, which showed that treating cells with Go-6976 partially prevented the development of insulin resistance in cells, which had been pretreated with high glucose and low dose insulin. Go-6976 is a widely used inhibitor of PKC, which is relatively selective for the conventional PKC isoforms, (cPKC α and β) and has much less affinity for the novel and atypical PKC-s. PKCβ is not expressed in NIH/hIR cells (mouse fibroblasts overexpressing the human insulin receptor) [9], and we were unable to detect it by immunoblotting in murine 3T3-L1 adipocytes, which are also fibroblast derived. PKCα was abundantly expressed in our cells, suggesting that we may have identified a candidate, which would be partially responsible for the development of glucose/insulin mediated insulin resistance. However, Go-6976 can also inhibit other enzymes including S6-kinase 1 [10], a substrate of mTORC1. It is also a potent *in vivo* inhibitor of protein kinase D (PKD) [11], [12]. The experiments described here led us to the conclusion that PKCα may not be involved in causing hyperglycemia-induced insulin resistance in adipocytes, and that at least some of the effects caused by Go-6976 may have been mediated via its inhibition of the mTORC1 pathway.

## Research Design and Methods

### Cell culture and glucose transport assay

3T3-L1 fibroblasts (ATCC 2001; cat #CL-173) were differentiated into adipocytes as previously described [3]. Cells were studied on days 8–12 from the initiation of the differentiation protocol, when >95% of the cells exhibited the adipocyte phenotype. They were maintained in Dulbecco's modified Eagle's medium (DMEM) containing 25 mM glucose and 10% fetal bovine serum (FBS). Before experiments the cells were exposed for 18 h to DMEM containing 1% FBS and either 5 mM or 25 mM glucose, with or without 0.6 nM insulin, as indicated. They were then serum and insulin deprived for 2 h, and after washing placed into Krebs Ringer bicarbonate/HEPES (KRBH) buffer without glucose for 10 min and then incubated with or without a maximally stimulating insulin dose (100 nM) for 15 min [3]. Glucose transport was assessed as the uptake of 2-deoxy-D-glucose (2-DOG) for 3 min as described [3] and normalized to protein concentration assessed using the Coomassie Protein Reagent (Pierce). In some experiments activators and/or inhibitors of PKC were added to the media before assay. The activator used was phorbol 12-myristate 13-acetate (PMA) (Calbiochem), the inhibitors used were Go-6976, a cell permeable inhibitor of PKC, which selectively inhibits PKCα and β1 isozymes, at nM concentrations, but does not affect the kinase activities of Ca2+ independent isozymes, δ, ε and ζ, even at µM concentrations (Cat. No. 365250), and bisindolylmaleimide I, Go-6850, which is a broader PKC inhibitor (Cat. No. 203290, both from Calbiochem). We also tested Ruboxistaurin (LY379196), kindly provided by Lilly Research Laboratories, which at the doses used (300 nM) is a highly selective inhibitor of PKCβ [8], [13].

### RNAi

To test the role of PKCα, PKCλ and mTORC1 pathway in our model of insulin resistance we investigated the effect of reducing their expression via transfection of mouse specific small interfering siRNA-s. We purchased SMARTpool reagents from Dharmacon against PKCα, PKCλ and against Raptor, each consist of a mixture of 4 unique siRNA-s developed against the targeted gene and are guaranteed to silence the RNA by >75%. For cells treated with non-targeting siRNA, we used Dharmacon's non-targeting siRNA pool of 4.

3T3-L1 adipocytes are notoriously difficult to transfect and until recently their successful siRNA transfection usually involved electroporation [14]. Recently, a technology using virus derived amphipatic peptides has been developed, that directly interact with nucleic acid cargos to form nanoparticles, which diffuse through plasma membranes and release their cargos inside the cell [15]. The technology has been adapted by the manufacturer (Panomics, Fremont, CA) to several cell lines, including 3T3-L1 adipocytes. We adopted the following protocol for transfection of the latter [6]. On day 6 after initiation of the differentiation protocol, cells were subcultured to a density of 2.5×10^4^ cells/cm^2^ and incubated overnight in DME containing 25 mM glucose and 10% FBS. Then FBS was removed and cells were washed with PBS and transfected with a final concentration of 40 nM non-targeting or targeting siRNA using the DeliverX Plus Kit (Panomics). Following 4 h incubation with the siRNA, FBS was added to a concentration of 10% and cells were incubated for an additional 18 h. Media was replaced with fresh DME containing 25 mM glucose and 10% FBS for 24 h. Cells were then incubated for 18 h in DME containing 1% FBS and 5 mM glucose or 25 mM glucose+0.6 nM insulin, with or without Go-6976 or rapamycin, then serum- and insulin-deprived for 2 h and glucose transport measured as described. The degree of knock-down was assessed by Western blotting for the respective protein.

### Western blots

Total post-nuclear cell lysates were prepared in lysis buffer as previously described [3], [6]. Cell extracts were separated on 7% or 12% SDS-PAGE and transferred to nitrocellulose. They were blocked for 1 h and then incubated overnight in blocking buffer containing one of the following: rabbit P-PKC α/β (Thr 638/641) antibody, P-Akt (Ser473) antibody, rabbit P-p70 S6kinase (Thr 389) antibody, rabbit P-S6 ribosomal protein (Ser 240/244) antibody or rabbit anti-Raptor antibody (all 1∶1000, each from Cell Signaling). Following washing, membranes were incubated in goat anti-rabbit IgG (1∶10,000, Jackson Immunoresearch) for 1 h, washed, developed with West Pico ECL Reagent (Pierce), exposed to film, and quantified by laser densitometry using a National Institute of Health Image Analyzer Program. Membranes that had been exposed to P-PKC α/β_2_ antibody were stripped and reanalyzed with an antibody to PKCα (1∶1000, Cell Signaling); while blots developed with anti-phospho- p70 S6kinase or with anti-phospho S6 ribosomal protein antibody were stripped and analyzed with mouse p70 S6kinase antibody (1∶200, Santa Cruz) or with rabbit anti-S6 ribosomal protein antibody (1∶1000, Cell Signaling), respectively. Finally, most gels were stripped and analyzed with rabbit β-tubulin antibody (1∶200, Santa Cruz) to document equal loading of the gel.

In some experiments the phosphorylation of Ser 24 of IRS-1 was tested, because this site is directly responsive to stimulation by PKCα [9]. The preparation of this antibody has been described [9]. Total post-nuclear cell lysates (1 mg protein) were immunoprecipitated with 2 µg of rabbit anti-IRS-1 antibody (Upstate/Chemicon) overnight at 4°C. Immunocomplexes were collected for 2 h with protein G Sepharose, washed and eluted with Laemmli's sample buffer for 10 min at 95°C. The eluates were separated by SDS-PAGE and exposed to P-IRS-1 (Ser 24) rabbit antibody, at a dilution of 1∶100 overnight at 4°C. Subsequent development with goat anti-rabbit IgG was as described above.

## Results and Discussion

### Go-6976 treatment enhances insulin-sensitivity in cultured adipocytes

**Figure 1:**
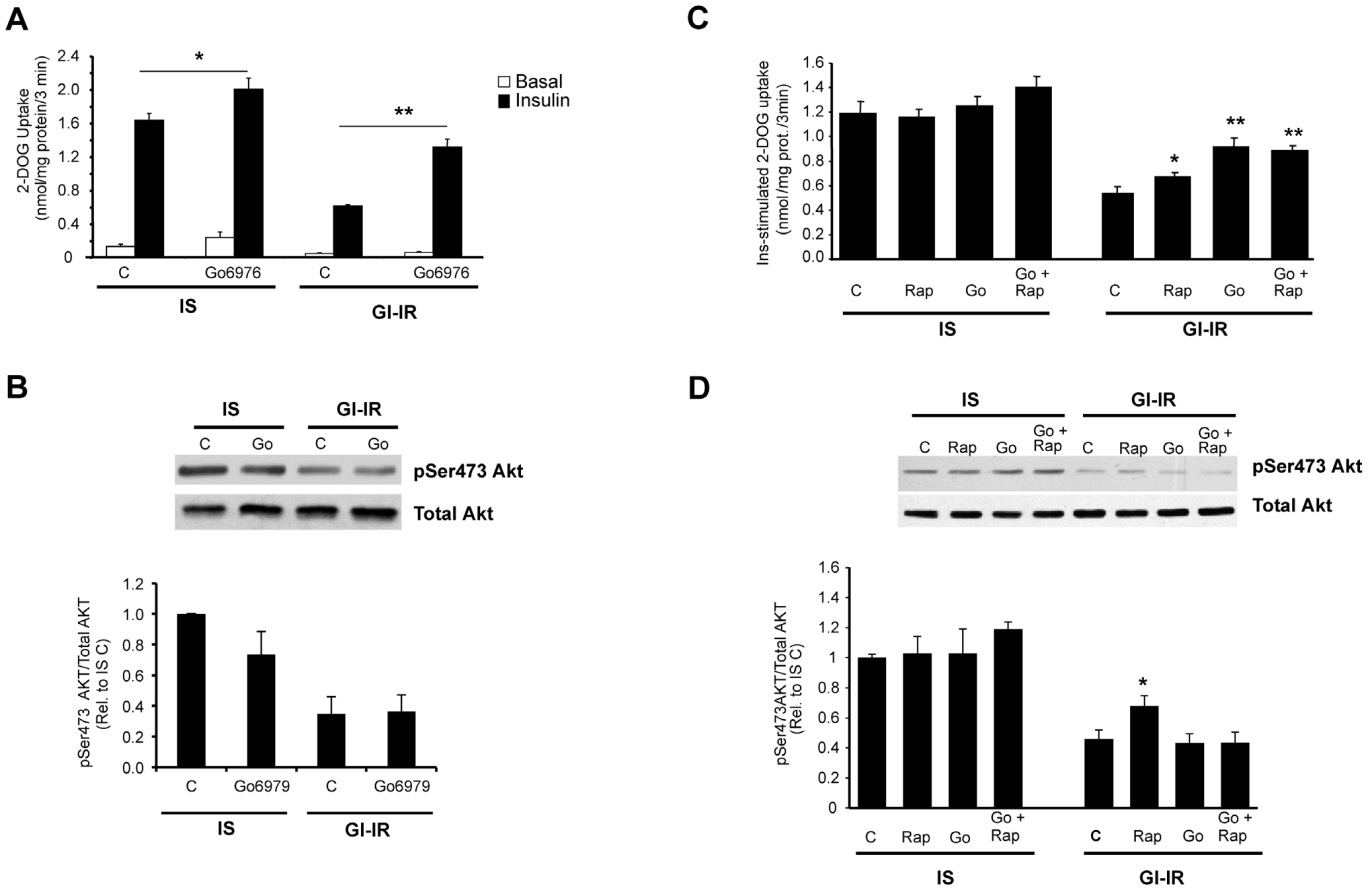
Treatment with Go-6976, reverses hyperglycemia-induced insulin resistance in adipocytes. **A.** Effect of Go-6976 on Glucose transport. Insulin sensitive (IS) Adipocytes were preincubated in 5 mM glucose while Glucose-induced insulin resistant, (GI-IR) were generated by preincubation in 25 mM glucose+0.6 nM insulin for 18 h. During serum and insulin deprivation for 2 h, adipocytes were received 0.3 mM Go-6976 or vehicle control during the last hour of incubation. Glucose (2DOG) uptake was determined in unstimulated (open bars, basal) or in insulin-stimulated (100 nM for 15 min; filled bars, Insulin) cells. **B.** Effect of Go-6976 on insulin-stimulated Akt phosphorylation. Adipocytes were treated as in A, following acute insulin stimulation, cell lysates were collected and both phospho-Akt and total AKT was measured. Quantification represents ratios of phosphoAKT/Total AKT protein expressed relative to ratios obtained in from control insulin sensitive (IS) adipocytes. **C.** The effects of rapamycin and Go-6976 on insulin-stimulated glucose transport. Adipocytes were treated as in A, except that only cells, which have been acutely stimulated with insulin, are shown. Go-6976 (0.3 mM) and rapamycin (100 nM) are added during the last hour of incubation. **D.** The effects of rapamycin and Go-6976 on insulin-stimulated Akt phosphorylation. Adipocytes were treated as in C, following acute insulin stimulation, cell lysates were collected and both phospho-Akt and total AKT was measured. Quantification represents ratios of phosphoAKT/Total AKT protein expressed relative to ratios obtained in from control insulin sensitive (IS) adipocytes. Statistical significance is indicated by *p<0.05, and ** = p<0.005 compared to controls with the same preincubation.

To generate hyperglycemia- and insulin-induced insulin resistant (GI-IR) adipocytes, 3T3-L1 adipocytes were preincubated for 18 h in culture media containing 25 mM glucose and supplemented with 0.6 nM insulin. In contrast, control ‘insulin-sensitive’ adipocytes (IS) were generated by preincubation with 5 mM glucose without insulin supplementation. Insulin-stimulated glucose uptake was then determined as detailed in materials and methods. As previously reported, preincubation in high glucose together with low dose insulin inhibited insulin-stimulated (p<0.001) glucose transport (**Fig. 1A**) confirming that these GI-IR adipocytes were indeed insulin resistant. To identify potential serine kinases that may be involved in mediating this insulin resistance and amenable to pharmacological intervention, we screened a number of kinase inhibitors. Amongst these, Go-6976 was identified as an inhibitor of cPKC isoforms [9] that significantly enhanced insulin-stimulated glucose transport both in the GI-IR adipocytes (by 114%, p<0.001) as well in IS adipocytes (by 23%, p<0.05). Although Go-6976 did not completely restore insulin stimulated glucose transport to control levels (p<0.04), the effects only required incubation with 0.3 µM inhibitor for 1 h immediately following the pretreatment and preceding determination of insulin sensitivity.

We have previously shown that the insulin resistance that develops in this model is associated with a marked inhibition of insulin-stimulated Akt activation [5], [7] and this was again observed in these experiments (**Fig. 1B**). However, Go-6976 treatment did not restore insulin-stimulated Akt activation, suggesting that the target of Go-6976 action is either downstream of, or occurs parallel to, Akt activation. This observation contrasts with our previous study in which exposure of GI-IR adipocytes for one hour to the mTOR inhibitor, rapamycin, partially restored the insulin resistant glucose transport, and simultaneously corrected the impaired activation of Akt [7]. To establish whether the effects of both serine kinase inhibitors (Go-6976 and rapamycin) on glucose transport were of equal magnitude, additive and/or synergistic, we performed additional parallel experiments. In this study there were no significant effects in control insulin sensitive (IS) adipocytes (**Fig. 1C** **)**. However, in GI-IR adipocytes, insulin-stimulated glucose transport was decreased by ∼60%. This was significantly reversed both by rapamycin and Go-6976 treatments when used individually, however, there was no evidence of any additive effect when these inhibitors were combined (**Fig. 1C**) suggesting that they may target the same pathway. Notably, insulin resistance was again not completely restored and insulin-stimulated AKT phosphorylation was also not restored with Go-6976 alone or combination with rapamycin (**Fig. 1D**).

### GI-IR adipocytes do not exhibit enhanced IRS-1 phosphorylation on Ser-24

**Figure 2:**
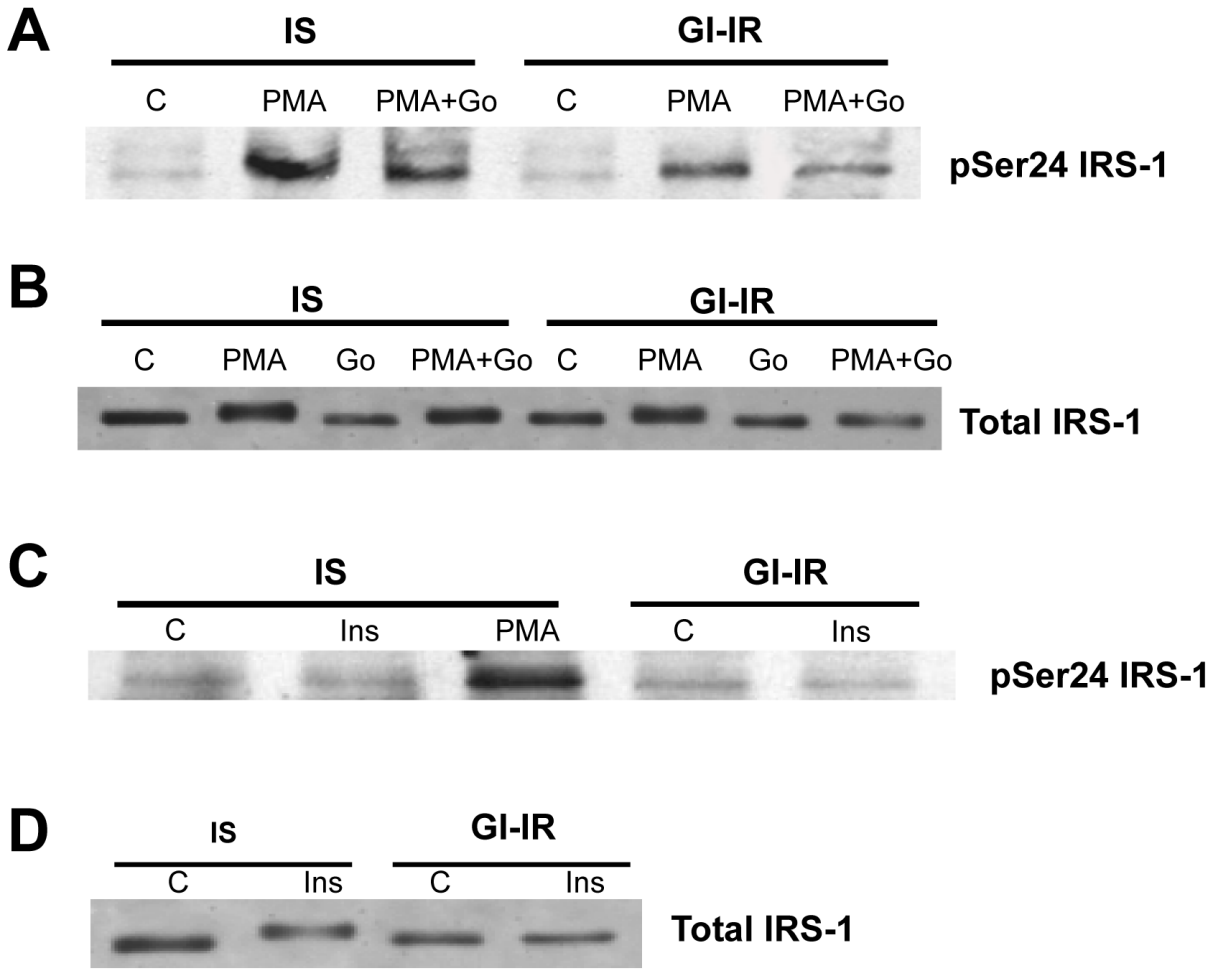
Hyperglycemia-induced insulin resistance does not promote pSer24 IRS-1, a target of cPKC. Adipocytes were preincubated for 18 h in 5 mM glucose (IS) or in 25 mM glucose+0.6 nM insulin (GI-IR). **A and B.** During the last hour, cells were exposed to either vehicle control (C), PMA (1 mM), Go-6976 (1 mM) or both. IRS-1 was immunoprecipitated from subsequent cell extracts and following gel separation western blotted with an anti-pSer 24 IRS1 (**A**) or anti-IRS1 (**B**), as described in Methods. **C.** pSer 24 IRS1 levels are unaffected by preincubation treatment and acute insulin stimulation. **D.** Total IRS1 protein exhibit a mobility shift following acute stimulation with 100 nM insulin for 15 min. All gels are representative of 3 similar experiments.

We recently reported that protein kinase C activators such as phorbol esters can promote insulin resistance through phosphorylation of Serine-24 on IRS-1 and that this was also inhibited by Go-6976 [9]. We therefore next investigated whether IRS-1 phosphorylation on Ser-24 was enhanced in GI-IR adipocytes. **Figure 2A** shows that 1 µM PMA can induce phosphorylation of IRS-1 on Ser-24 in IS adipocytes and this coincided with a mobility shift in total IRS-1 (**Fig. 2B**). Inclusion of Go-6976 (1 µM) with the PMA reduced both Ser-24 phosphorylation of IRS1 and the reduction in total IRS-1 mobility (**Fig. 2A and 2B**). This is consistent with similar findings reported for NIH3T3 cells [9]. However, in GI-IR adipocytes, basal IRS-1 Ser-24 phosphorylation was unaltered and the response to PMA with or without Go-6976 was similar to IS adipocytes. **Figure 2C** also shows that acute stimulation with insulin alone (100 nM, 15 min) also did not induce Ser-24 phosphorylation in the control (IS) or in the insulin resistant (GI-IR) adipocytes. However, acute insulin was sufficient to induce a mobility shift in total IRS-1 at least in IS adipocytes (**Fig. 2D**). These data suggest that PKCα-mediated phosphorylation of Ser24 on IRS-1 is not involved in hyperglycemia-induced insulin resistance.

### GI-IR adipocytes do not exhibit altered PKCα expression or activating phosphorylation

**Figure 3:**
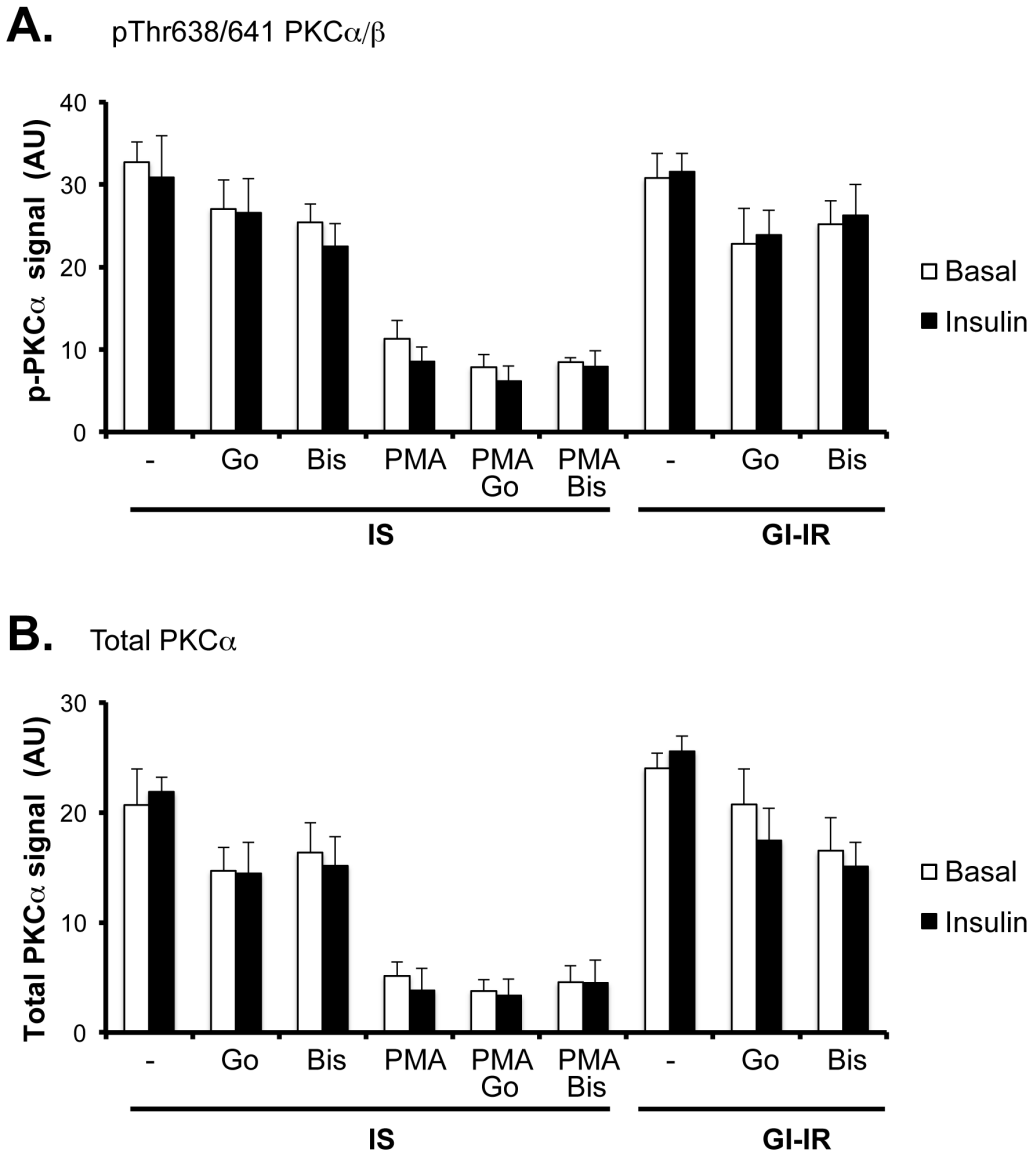
Phosphorylated PKCalpha and total PKCalpha are not altered by hyperglycemia-induced insulin resistance. Adipocytes were preincubated for 18 h in 5 mM glucose (IS) or in 25 mM glucose+0.6 nM insulin (GI-IR). During the last 2 hours cells were exposed to either vehicle control or PMA (1 mM), or during the last hour with Go-6976 (1 mM, Go) or bis-indolylmaleimide (1 mM, Bis). Acute insulin stimulation (black bars) or non-stimulation (white bars) were performed for 15 min with or without 100 nM insulin. Cells were lysed, proteins extracted and subjected to western blotting with antibodies to either phosphorylated pThr638/641 PKCalpha/beta (**A**) or to Total PKCalpha (**B**). Quantification was determined as described in Methods. The graph data represents means +/− SE of 6 observations.

Since Go-6976 is widely reported to be an inhibitor of cPKCs (PKCα/β), and cPKCs have themselves been implicated in other models of insulin resistance, we next investigated their presence and putative regulation in GI-IR adipocytes. We were unable to detect PKCβ expression in our cells (data not shown). However, PKCα protein was detected, as was pThr638/641- PKCα, an indicator of active PKCα levels (**Fig. 3** **and Fig. S1**). Importantly, neither the preincubation conditions (low glucose versus high glucose plus low dose insulin) nor acute stimulation with high dose insulin affected either the total amount or the phosphorylation of PKCα significantly (**Fig. 3** **and Fig. S1**), suggesting that PKCα is not involved in this model of insulin-resistance. However, the addition of Go-6976 significantly reduced the expression of PKCα (**Fig. 3B**, p<0.001). Nonetheless, there was no difference in the effect of Go-6976 between IS adipocytes (preincubated in 5 mM glucose) and GI-IR adipocytes. The effect of Go-6976 on the phosphorylation of PKCα showed the same trend as that of total PKCα expression. Although the effect was less pronounced, it was significant by analysis of variance (p<0.01). In contrast, incubation of adipocytes for two hours with 1 µM PMA markedly reduced the expression and phosphorylation of PKCα (**Fig. 3**, p<0.001). The ratio of phospho-PKC∶Total PKCα was not significantly affected suggesting that PMA-stimulated effects involved primarily PKCα degradation. Indeed it is well known that sustained exposure to PMA promotes activation followed by degradation of cPKC [9]. Addition of Go-6976 or bisindolylmaleimide I for 1 hour to PMA pretreated cells did not change the PMA effect. The surprising lack of effect of the PKC inhibitors likely reflects the fact that adipocytes were first treated with PMA for 1 hr followed by a further 1 hr with inhibitors (Go-6976 or of bisindolylmaleimide I) together with PMA. An alternative commonly used albeit preventative approach, would have been to pretreat adipocytes with these inhibitors prior to and during PMA exposure. This approach may have yielded different results with respect to the effects of PMA.

### PKCα knock-down does not alter insulin resistance in GI-IR adipocytes

**Figure 4:**
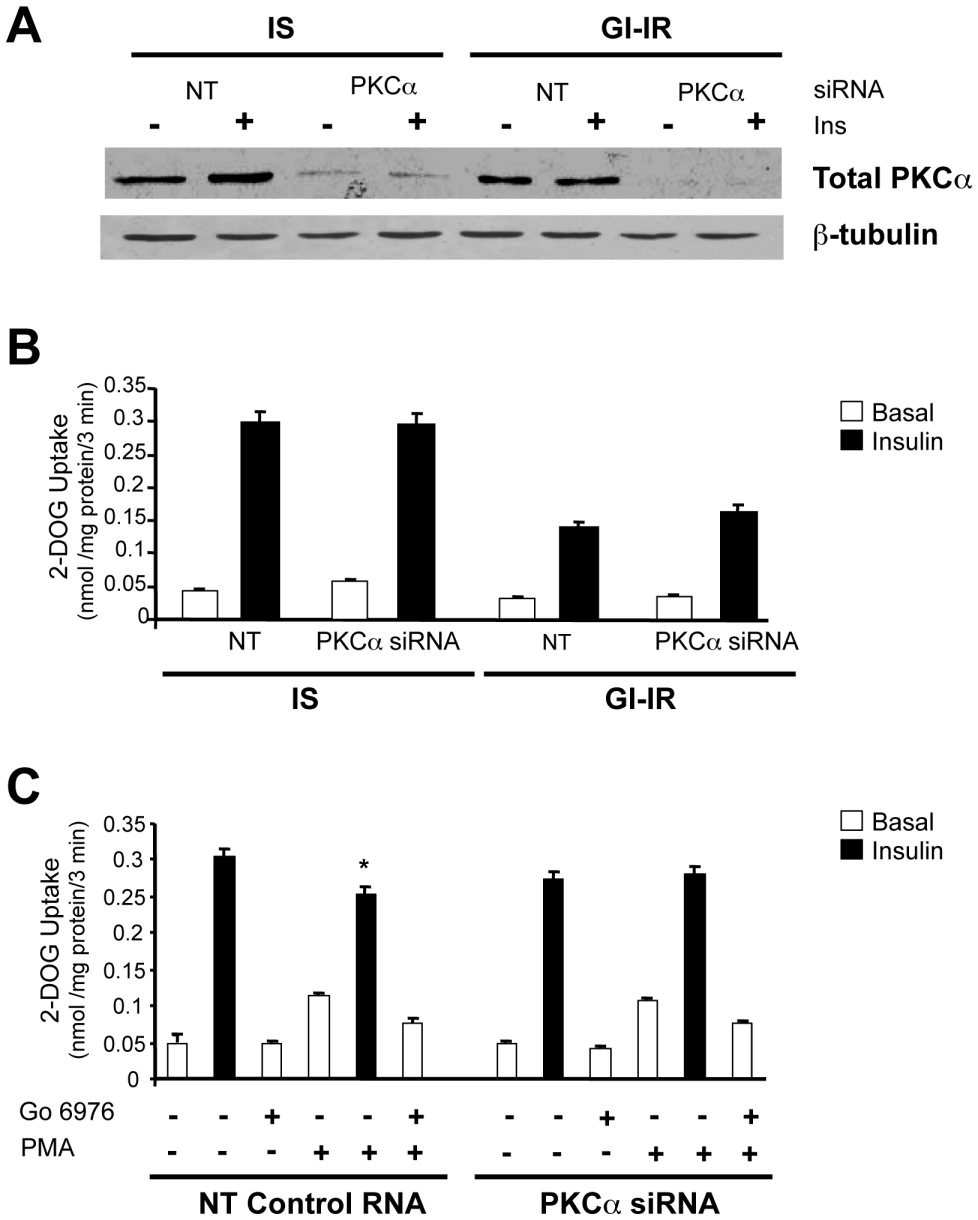
PKCalpha knock-down does not prevent the development of hyperglycemia-induced insulin resistance. **A.** The expression of PKCαlpha is reduced (by 75–78%) in adipocytes treated with anti-PKCαlpha siRNA compared with cells infected with non-targeting (NT) siRNA. **B.** Experiment designed was as described for Fig. 1 except cells were treated with indicated siRNA prior to glucose uptake determination. **C.** PMA-induced insulin resistance is lost in cells infected with anti-PKCαlpha siRNA. Adipocytes were infected with non-targeting or with anti-PKCalpha siRNA, then treated with or without PMA (1 µM, 1 h) followed by acute insulin stimulation and glucose uptake determined. Statistical significance is indicated by *p<0.005 vs. no PMA.

To further assess the role of PKCα, we tested the effect of its knock-down on the development of insulin resistance in our model. Cells transfected with siRNA against PKCα were compared to cells transfected with non-targeting RNA and tested 72 h after transfection. PKCα expression at this time was reduced by 75–78% as judged by Western blots (**Fig. 4A**). As shown in **Fig. 4B**, glucose transport and the development of insulin resistance were not affected by the PKCα knock-down in either IS or GI-IR adipocytes. To further assess whether PKCα knock-down had any effect on insulin-stimulated glucose transport, we compared the effects of PMA and Go-6976, in the presence or absence of insulin on glucose transport in IS adipocytes (**Fig. 4C**). PMA (1 µM, 1 h) stimulated glucose transport (>2-fold over basal) in both the PKCα knock-down and the non-targeted controls and the addition of Go-6976 to PMA partially blocked this effect. The only significant difference between the PKCα knock-down and the non-targeting controls was that in the latter PMA inhibited maximally insulin-stimulated glucose transport by ∼17% (p<0.005) while this did not occur in the PKCα knock-down cells. The data suggests that while PKCα does play a role in the effect of PMA on glucose transport in these cells, confirming previous observations [16], it does not seem to participate, in the development of hyperglycemia-induced insulin resistance in GI-IR adipocytes.

### PKCβ inhibition does not reverse insulin resistance in GI-IR adipocytes

Although we had no evidence that PKCβ is expressed in our 3T3-L1 adipocytes, we also tested whether incubation with a specific inhibitor of this isoform would alter the insulin resistant glucose transport in our experiments. Ruboxistaurin had no effect on glucose transport under any of the conditions tested and failed to affect the insulin response of glucose transport (n = 5, from two experiments, **Fig. S2**).

### Effect of PKCλ knock-down on insulin-stimulated glucose transport

**Figure 5:**
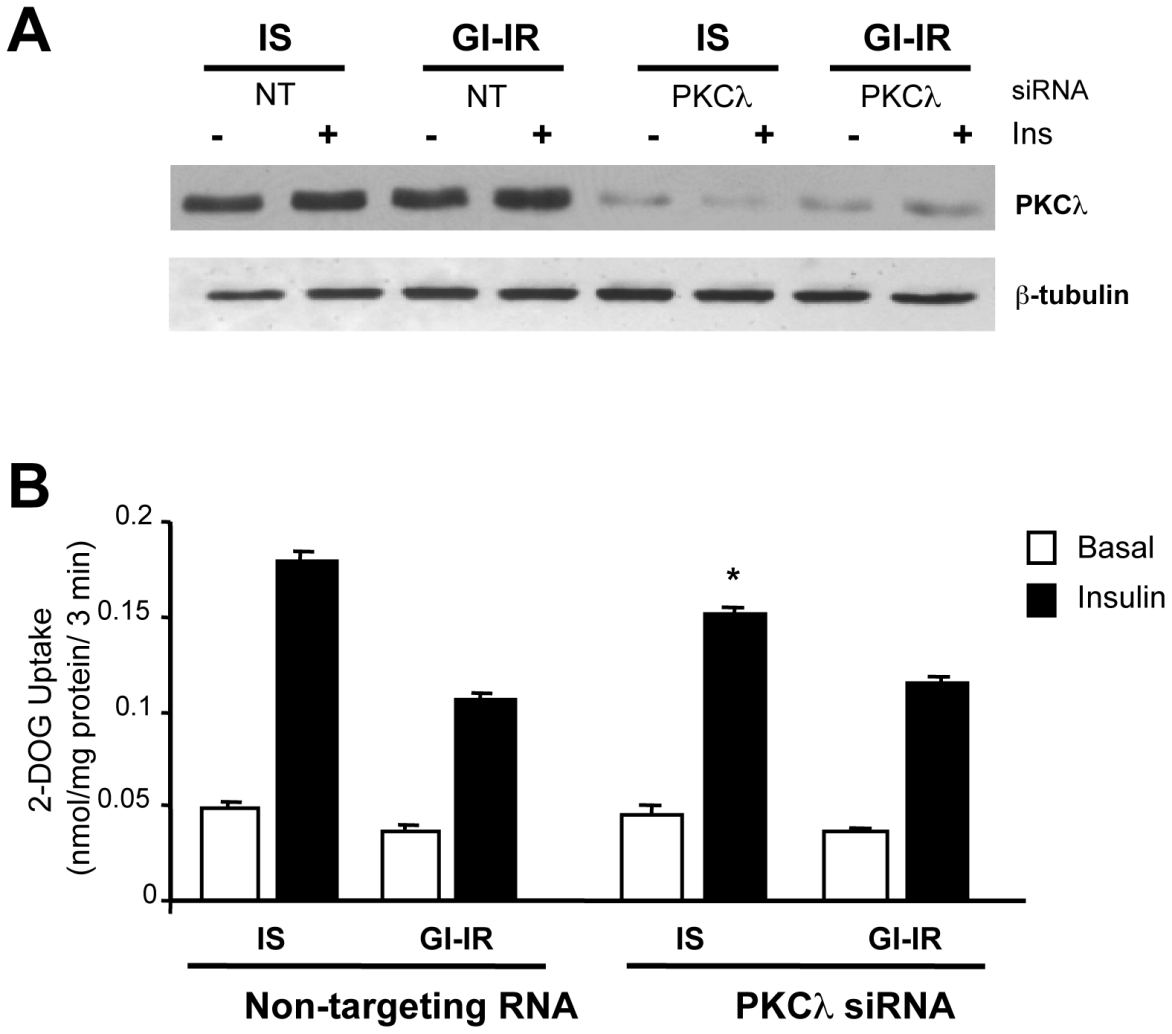
The effect of PKCλ knock-down on hyperglycemia-induced insulin resistance. **A.** Western blot showing depletion (by 84%) of PKCλ in cells treated with anti-PKCλ siRNA as compared to cells treated with NT control RNA. **B.** cells preincubated in 5 mM glucose (IS) vs. 25 mM glucose+0.6 nM insulin (GI-IR) and then stimulated (the black bars) or not (basal) with acute, 100 nM insulin. Knock-down of PKCλ mRNA decreased the insulin-stimulation of glucose transport by ∼20%, in IS adipocytes, but had no additional effect in GI-IR adipocytes. (*p<0.001, n = 15).

In view of the elegant demonstration that atypical PKC's are required for insulin-stimulated glucose transport in 3T3-L1 adipocytes [17] we also investigated the role of PKCλ (the a-PKC expressed in adipocytes) in our model. PKCλ expression was decreased by 84% in cells transfected with mouse specific small interfering si-RNA against PKCλ (**Fig. 5A**). In cells incubated in 5 mM glucose the insulin effect on glucose transport was decreased by 20% in RNAi treated adipocytes (**Fig. 5B**, p<0.001) compared to cells treated with a non-targeting RNA. Our data qualitatively reproduces previously published observations [17]. However, it is of interest, that in GI-IR adipocytes, knock-down of PKCλ had no additional inhibitory effect.

### Go-6976 affects the phosphorylation of S6 ribosomal protein

**Figure 6:**
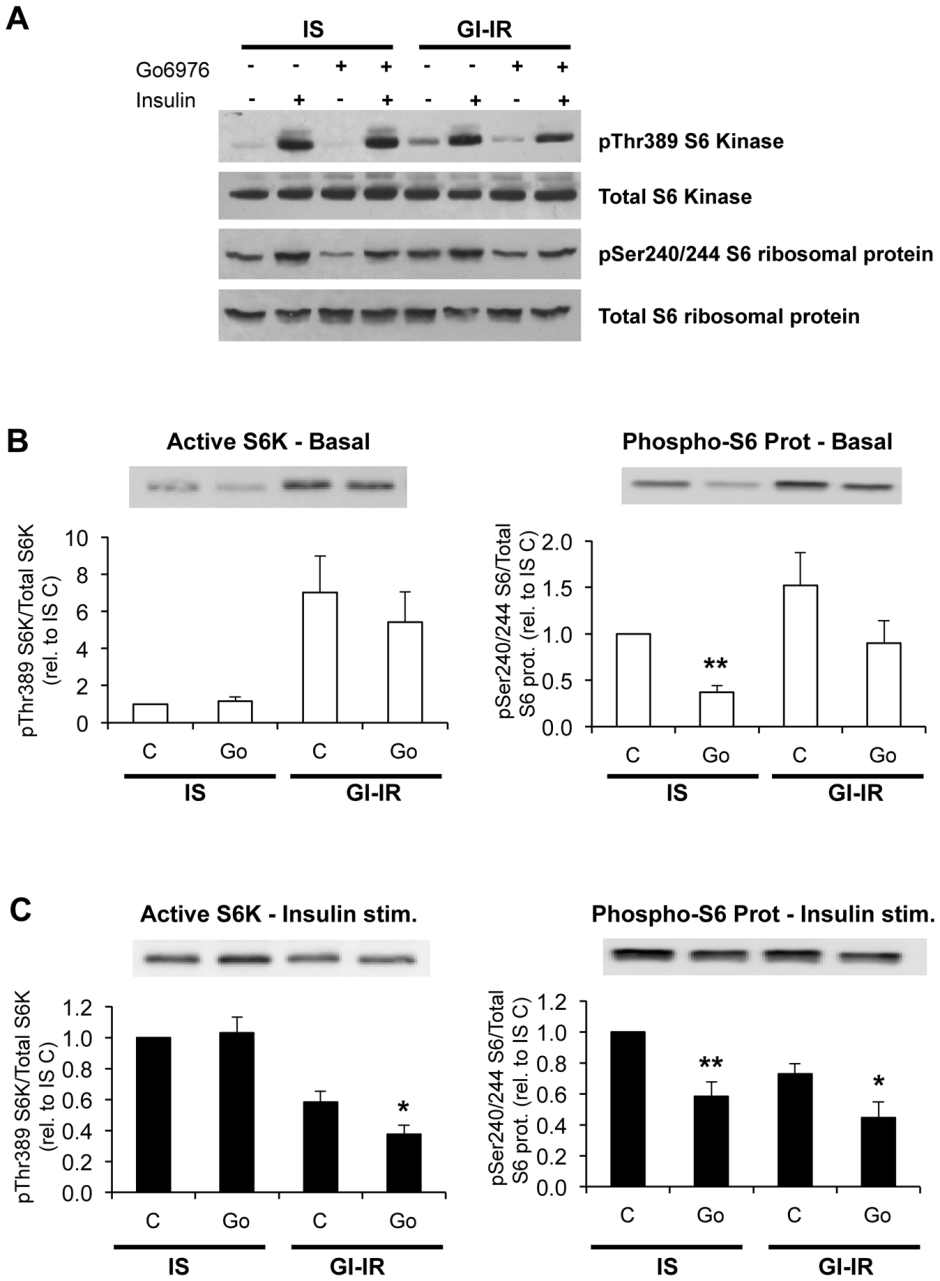
Effect of Go-6976 on S6 Kinase activity. Adipocytes were preincubated for 18 h in 5 mM glucose (IS) or in 25 mM glucose+0.6 nM insulin (GI-IR). **A.** Acute insulin treatment (100 nM for 15 min) stimulated activating phosphorylation of S-6 kinase (8–10-fold) and of its substrate S-6 ribosomal protein (4–5-fold). Under basal conditions, GI-IR adipocytes exhibited enhanced S6K phosphorylation. For quantification purposes, unstimulated (**B**) and acute insulin-stimulated (**C**) samples were also analysed on separate gels with exposure to times adjusted to within linear ranges. Under these conditions it is evident that the acute insulin stimulated S6K activity was decreased in the GI-IR cells. This was further reduced by treatment with Go-6976 (1 mM for 1 h). Statistical significance is indicated by *p<0.05 vs. control without Go-6976, **p<0.001. n = 9.

In view of the above findings we next investigated whether Go-6976 may reverse the development of insulin resistance by inhibiting enzyme(s) other than cPKC but downstream of AKT. Indeed, *In vitro* studies using recombinant peptides have suggested that Go-6976 can interact with additional kinases, including p70 S6 kinase-1 [10], which we previously reported to be involved in our model of insulin resistance [7]. We therefore next measured the insulin-stimulated activation of S6 kinase-1 (i.e. pThr389-S6K∶Total S6K ratio) and the phosphorylation of its substrate, S6 ribosomal protein (on Ser 240/244). As reported previously [7], acute insulin treatment of IS adipocytes markedly stimulated the activation of S6 kinase (8–10-fold) and increased the expression of phosphorylated S6 ribosomal protein (3–5-fold, **Fig. 6A**). In GI-IR adipocytes, basal levels of active S6 kinase-1 and S6 ribosomal protein phosphorylation was significantly elevated (**Fig. 6B**) and Go-6976 treatment prevented direct correlation between active S6K and phosphorylated S6 ribosomal protein (**Fig. 6B**). However, insulin-stimulated activation of this pathway was reduced in GI-IR adipocytes, and Go-6976 treatment further reduced both active S6K and S6 ribosomal protein phosphorylation (**Fig. 6C**). These data suggest that p70 S6 kinase-1 is a candidate target for Go-6976 action in adipocytes.

### Raptor knock-down enhances insulin-stimulated glucose transport in GI-IR adipocytes but is additive with Go-6976

**Figure 7:**
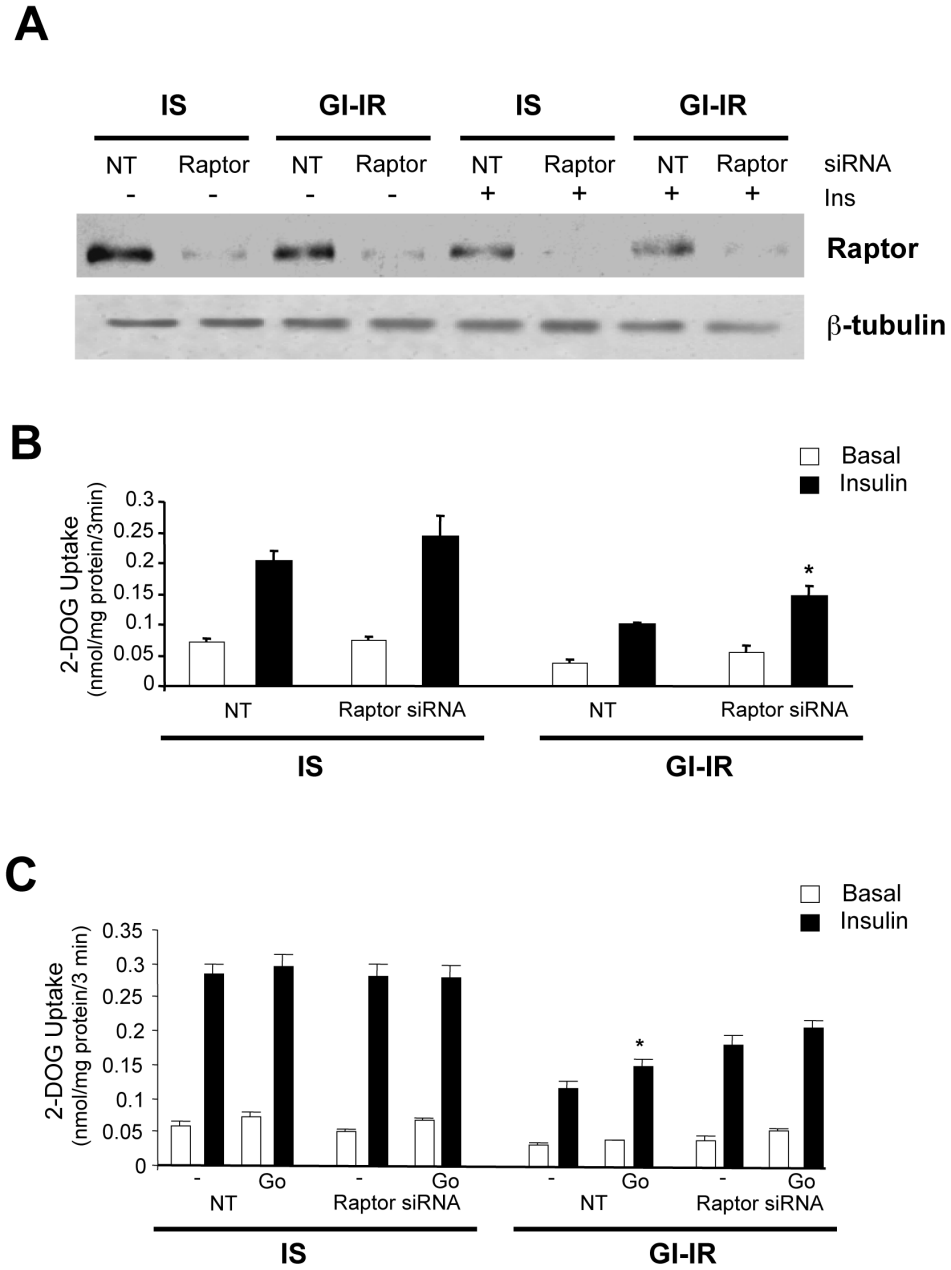
Raptor knock-down enhances the effect of insulin on glucose transport in insulin resistant cells. **A** Raptor expression was decreased by 76% in adipocytes treated with anti-Raptor siRNA compared to cells treated with NT RNA. **B** Insulin-stimulated glucose transport is enhanced by ∼40% in hyperglycemia-induced insulin resistant (GI-IR) Raptor knock-down cells (* = p<0.005 vs. NT). **C** Go-6976 enhances insulin stimulated glucose transport only in the insulin resistant NT cells (* = p<0.03), but not in the Raptor knock-down cells.

Since S6K-1 is a downstream target of mTOR-associated proteins such as raptor we next investigated whether insulin-stimulated glucose transport was altered in our model when Raptor was knocked-down. Raptor expression decreased by 76% in the cells treated with siRNA for Raptor (**Fig. 7A**). In IS adipocytes, insulin stimulated glucose transport was not significantly different in the Raptor knock-down cells as compared to the non-targeted controls (**Fig. 7B**). However, in GI-IR adipocytes, insulin-stimulated glucose transport increased by 40% in the Raptor-deficient cells, a significant effect (**Fig. 7B**, p<0.04). We next compared the response to Go-6976 of glucose transport between non-targeted and knock-down cells (**Fig. 7C**). In IS adipocytes, there was no difference either in basal or acute insulin stimulated glucose transport between control and Raptor deficient cells and the addition of 1 µM Go-6976 also had no effect. However, in GI-IR adipocytes, Go-6976 significantly increased insulin-stimulated glucose transport in the non-targeted cells (p<0.03) but was unable to further improve glucose transport in Raptor knock-down cells (**Fig. 7C**). Again, without Go-6976 treatment the insulin-stimulated glucose transport was greater (p<0.005) in the Raptor knock-down cells than in the non-targeted controls (**Fig. 7C**). These data suggest that the mTORC1 pathway indeed plays an important role in GI-IR adipocytes and that Go-6976 alleviates this by targeting inhibition of mTORC1 dependent pathway(s).

In summary, the major message of this paper is that the mTORC1 pathway does play a role in eliciting high glucose/low dose insulin mediated insulin resistance (GI-IR) in cultured adipocytes, while we were unable to detect any significant role of cPKC with the methods used here. This is important, because cPKCs are thought to be important mediators of hyperglycemia-associated pathology and experiments with the widely used cPKC inhibitor Go-6976 initially suggested that cPKC-s might play a major role in high glucose-induced insulin resistance in adipocytes. However, our data clearly indicate that Go-6976 exerts its major effects by inhibiting mTORC1 dependent pathways in the GI-IR adipocytes. It is worth noting that this study has focused on one *in vitro* model of hyperglycemia-induced insulin resistance in murine adipocytes. As such, our findings do not rule out the possibility that other PKC's, novel or atypical, may play a role in the development of insulin resistance induced by other mediators on in other cellular systems. Indeed, there is ample evidence that lipid/FFA-induced insulin resistance is associated with activation of PKCθin rodent skeletal muscle and PKCδ and -β in human muscle. Furthermore, in liver lipid-induced insulin resistance has been linked to PKCε activity (reviewed in [18]). Hence, additional studies are warranted before a significant clinical impact can be assigned to our findings. Such studies should elucidate the molecular target of Go-6976 action and confirm that similar therapeutic effects also occur in human adipocytes and other insulin target tissues. Such mechanistic insights will be crucial in informing future targeted therapeutics aimed at treating patients with hyperglycemia and IR while avoiding unanticipated side effects.

Nonetheless, our findings highlight the fact that caution should be exercised when interpreting mechanistic data from pharmacological studies alone because potentially misleading conclusions can be drawn from use of very modest doses a ‘specific’ pharmacological inhibitor in cultured cells. That Go-6976 may not be as specific as advertised has been reported previously, where its inhibition of recombinant S6 kinase 1 has been demonstrated [10]. By combining both targeted pharmaceuticals with specific gene modulation we have demonstrated that a more reliable and powerful approach can be taken to dissect complex signaling pathways.

## Supporting Information

Figure S1 **Phosphorylated PKCalpha and total PKCalpha are not altered by hyperglycemia-induced insulin resistance.** Adipocytes were treated as described for Figure 3. Representative western blots are shown for basal (A) and Insulin stimulated (B) samples. Quantification is shown in Figure 3.
(PDF)

Figure S2 **The PKCbeta inhibitor, Ruboxistaurin does not affect insulin resistance in 3T3-L1 adipocytes.** Cells were preincubated in 5 mM glucose (IS) or in 25 M glucose+0.6 nM insulin (GI-IR) and then acutely stimulated with (the black bars) or without (the white bars) 100 nM insulin for 15 min. Some samples were also supplemented with 300 nM Ruboxistaurin (Rbxn) for 18 h or for 2 h, before insulin stimulation. Ruboxistaurin did not affect insulin stimulation of glucose transport under any of the conditions tested (n = 6).
(PDF)
