# Supplementary figures and images for: Go-6976 Reverses Hyperglycemia-Induced Insulin Resistance Independently of cPKC Inhibition in Adipocytes
Source: PLoS One. 2014 Oct 15;9(10):e108963. doi: 10.1371/journal.pone.0108963 (PMC4198081; doi:10.1371/journal.pone.0108963)

## A. Basal

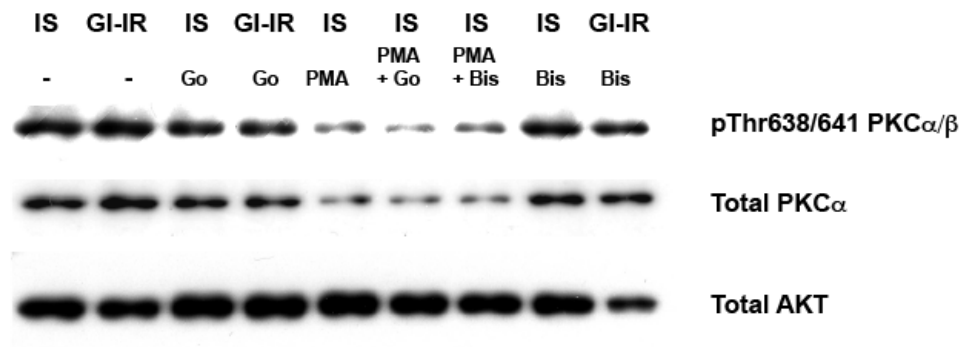

## B. Insulin stimulated

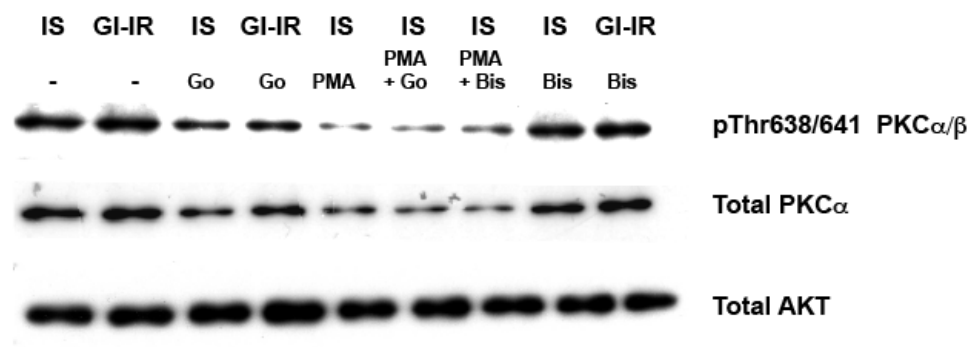

Supplement: Figure S1 — Phosphorylated PKCalpha and total PKCalpha are not altered by hyperglycemia-induced insulin resistance. Adipocytes were treated as described for Figure 3. Representative western blots are shown for basal (A) and Insulin stimulated (B) samples. Quantification is shown in Figure 3. (PDF) [file pone.0108963.s001.pdf]

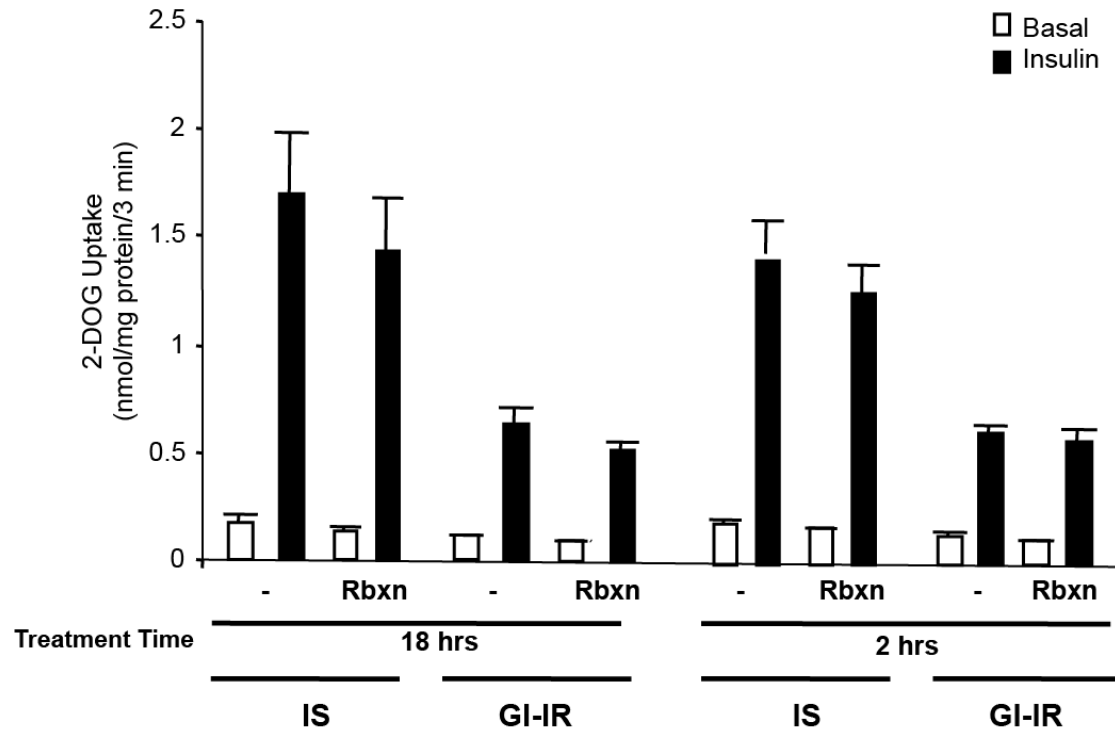

Supplement: Figure S2 — The PKCbeta inhibitor, Ruboxistaurin does not affect insulin resistance in 3T3-L1 adipocytes. Cells were preincubated in 5 mM glucose (IS) or in 25 M glucose+0.6 nM insulin (GI-IR) and then acutely stimulated with (the black bars) or without (the white bars) 100 nM insulin for 15 min. Some samples were also supplemented with 300 nM Ruboxistaurin (Rbxn) for 18 h or for 2 h, before insulin stimulation. Ruboxistaurin did not affect insulin stimulation of glucose transport under any of the conditions tested (n = 6). (PDF) [file pone.0108963.s002.pdf]
